# Analysis of microbial diversity in the feces of *Arborophila rufipectus*

**DOI:** 10.3389/fmicb.2022.1075041

**Published:** 2023-02-02

**Authors:** Xiaoping Ma, Junshu Li, Benping Chen, Xinni Li, Zhenwen Ling, Shenglin Feng, Sanjie Cao, Zhicai Zuo, Junliang Deng, Xiaobo Huang, Dongjie Cai, Yiping Wen, Qin Zhao, Ya Wang, Zhijun Zhong, Guangneng Peng, Yaozhang Jiang, Yu Gu

**Affiliations:** ^1^Key Laboratory of Animal Disease and Human Health of Sichuan Province, College of Veterinary Medicine, Sichuan Agricultural University, Chengdu, China; ^2^Authority of Administration, Sichuan Laojunshan National Nature Reserve, Yibin, China; ^3^Department of Bioengineering, Sichuan Water Conservancy Vocational College, Chengdu, China; ^4^College of Life Sciences, Sichuan Agricultural University, Chengdu, China

**Keywords:** gut microbiota, high-throughput sequencing, *Arborophila rufipectus*, feces, potential pathogens, 16S rRNA

## Abstract

**Introduction:**

Intestinal microbiota composition plays a crucial role in modulating the health of the host. This evaluation indicator is very sensitive and profoundly impacts the protection of endangered species. Currently, information on the gut microbiota of wild birds remains scarce. Therefore, this study aimed to describe the gut microbial community structure and potentially, the pathogen composition of wild *Arborophila rufipectus*.

**Methods:**

To guarantee comprehensive data analysis, we collected fecal samples from wild *A. rufipectus* and *Lophura nycthemera* in their habitats for two quarters. The 16S rRNA gene was then sequenced using high-throughput sequencing technology to examine the intestinal core microbiota, microbial diversity, and potential pathogens with the aim of determining if the composition of the intestinal microflora varies seasonally.

**Results and Discussion:**

The gut microbiota of *A. rufipectus* and *L. nycthemera* primarily comprised four phyla: Proteobacteria (45.98%), Firmicutes (35.65%), Bacteroidetes (11.77%), and Actinobacteria (3.48%), which accounted for 96.88% of the total microbial composition in all samples. At the genus level, core microorganisms were found, including *Shigella* (10.38%), *Clostridium* (6.16%), *Pseudomonas* (3.03%), and *Rickettsiella* (1.99%). In these genera, certain microbial species have been shown to be pathogenic. This study provides important indicators for analyzing the health status of *A. rufipectus* and formulating protective measures.

## 1. Introduction

*Arborophila rufipectus* is a mountain partridge that is a member of the pheasant family. It is a medium-sized partridge with rich colors and a body length of approximately 30 cm. Currently, it is only distributed in southwest China (He et al., 2009). It is a national first-class key protected animal in China (IUCN, 2016) due to its small range, very small population size, and severely fragmented habitat (Dai et al., 1998). Anatomically, *A. rufipectus* has an asthenia and much longer tarsometatarsal, with rounded and short wings compared to other birds. Thus, they are poor fliers with low aerial maneuverability (Yan et al., 2017). Their abilities and nature make them more vulnerable to human activities and natural enemy predators.

The gastrointestinal tract is a series of hollow organs populated with billions to trillions of microorganisms; their number exceeds that of the vertebrate’s own cells (Xu et al., 2015). Like other vertebrates, wild birds have hundreds and thousands of microbial species and densities of up to 1,011 CFU/g in their intestinal tract (Barnes, 1979). With significant advances in high-throughput sequencing technologies, studying gut microbial populations in wild birds has become economically feasible (Knutie and Gotanda, 2018). Through research, these gut microbiotas are increasingly recognized to play crucial roles in their hosts’ physiology, nutrition, metabolism, disease tolerance, and immune function (Umetsu et al., 2002; Carroll et al., 2011). Imbalances in the gut microbiota can cause various diseases (Dominguez-Bello et al., 2019). In the existing studies on the intestinal microflora of birds, the gut microbiota of birds is mainly composed of the following four phyla, Actinobacteria, Bacteroidetes, Firmicutes, and Proteobacteria (Waite and Taylor, 2014).

Current research on the gut microbiota of birds mainly focuses on poultry, such as chickens, ducks, geese, and turkeys (Waite and Taylor, 2015). Little research has been conducted on wild birds, whose flight ability gives them strong migration and dispersal abilities, higher metabolic rates, and affects their food and habitat, all of which can in turn affect gut microbial groups (Guan et al., 2020). However, data on the gut microbiota of *A. rufipectus* are scarce. We believe intestinal microflora analysis can provide useful insights into the health status of *A. rufipectus* and the microorganisms that affect the species. It has significant potential for informing conservation decisions for *A. rufipectus* (Trevelline et al., 2019). We settled on the feces of *Lophura nycthemera* as our sample as *L. nycthemera* is the companion bird of *A. rufipectus*. Furthermore, their living space is highly overlapped, and their food habits are similar. They also both mainly live on land, have weak flying abilities, and belong to the pheasant family. Moreover, we mainly used *L. nycthemera* as a species control, and it was positioned as a variable species to study the diversity relationship of intestinal flora in different bird species that share the same habitat.

This study aimed to describe the gut microbial community structure and potential pathogen composition of wild *A. rufipectus* using high throughput 16S rRNA gene sequencing. Additionally, we compared the gut microbiomes of *L. nycthemera* to determine similarities and differences between these species. The results of this study will help identify the core gut microbiota in *A. rufipectus*, improve our understanding of gut microbiota-mediated conservation of this bird species, and provide baseline information for developing targeted protective measures.

## 2. Materials and methods

### 2.1. Sampling

Samples were collected from the Sichuan Laojunshan Mountain National Nature Reserve, China (103°57′–104°04′N, 28°39′–28°43′E). We collected 10 fresh fecal samples from *L. nycthemera* (group A) and *A. rufipectus* (group B) between April 2021 and June 2021 (summer) and labeled them as group A/B (A1–A10, B1–B10). Another 10 fresh fecal samples from *A. rufipectus* were collected between July 2021 and September 2021 (autumn) and were labeled as group C (C1–C10). The feces were stored in sterile centrifuge tubes, avoiding ground contact during collection and using separate tools for each sample. Samples were collected at least 5 m apart to ensure that feces were excreted from different individuals. During the fieldwork, all samples were frozen in a portable freezer at −20°C. Samples were stored in liquid nitrogen and sent to the laboratory for subsequent processing. The detailed data collected for each sample are shown in Supplementary Table S1. *Lophura nycthemera*’s range of activities increased in autumn, and no stool samples that met the requirements were found within the stool collection range we set.

### 2.2. Isolation and identification of bacteria

Feces were inoculated on MacConkey (MAC) agar, Salmonella Shigella (SS) agar, and blood agar mediums using a disposable aseptic inoculation ring. All plates were incubated in an incubator (35°C), and the bacterial growth was monitored at 12-h intervals. We selected bacterial colonies of different shapes and colors for isolation until a single colony was obtained by purification. We used the TIANamp Bacteria DNA Kit (DP302; TIANGEN, Beijing, China) to extract bacterial DNA. PCR amplification of the V1-V9 region was performed using universal primers: 27f (5′-TACGGYTACCTTGT-TACGACTT-3′) and 1492R (5′-AGAGTTTGATCMTGGCTCAG-3′). The PCR mixture consisted of 2.0 μl DNA template, 9.0 μl 2 × Taq PCR Super Master Mix and 2.0 μl forward and reverse primers, respectively, and 10 μl ddH_2_O. The cycling conditions were as follows: initial denaturation at 94°C for 10 min; 30 cycles run at 94°C for 30 s, 55°C for 30 s, and 72°C for 2 min; and final extension at 72°C for 5 min. PCR products were sent to Sangon Biotech (Shanghai, China) for sequencing, and NCBI BLAST^1^ was performed on the sequencing data. The data were then compared with the sequence data available in the GenBank data of the NCBI, and MEGA5 was then used to build trees for contrastive sequencing data.

### 2.3. DNA extraction

The CTAB method was selected to extract total DNA from fecal samples, and agarose gel electrophoresis was used to confirm the quality of DNA extraction (Bansal et al., 2018). Finally, an ultraviolet spectrophotometer was used to quantify DNA. The total DNA was stored at −80°C and sent to LC-Biotechnology Co., Ltd. (Hang Zhou, China) to perform the polymerase chain reaction.

### 2.4. PCR amplification and 16S rDNA sequencing

Specific primers (16S V3-V4: 341F:5′-CCTACGGGNGGCWGCAG-3′, 805R: 5′-GACTACHVGGGTATCTAATCC-3′; Logue et al., 2016) were used to magnify the hypervariable region (V3–V4 regions) of the 16S rRNA gene sequence. The total amplification volume of PCR was 25 μl: 2.5 μl of forward and reverse primers, 12.5 μl of Phusion Hot Start Flex 2X Master Mix, 50 ng of template DNA, and water added to make 25 μl. The PCR cycling conditions were an initial denaturation at 98°C for 30 s, denaturation at 98°C for 10 s, annealing at 54°C for 30 s, an extension at 72°C for 45 s of 35 cycles, and a final extension at 72°C for 10 min. The PCR products were verified using 2% agarose gel electrophoresis. The purified PCR products were evaluated with the Library Quantification Kit of Agilent 2100 Biological Analyzer (Agilent, Santa Clara, CA, United States) and Illumina (Kapa Biosciences, Woburn, MA, United States). The qualified library concentration was above 2 nM. Using NovaSeq 6000 Sequencer 2 × 250 bp double-ended sequencing, the corresponding reagent was NovaSeq 6000 SP.

### 2.5. Data analysis

In accordance with the LC-Bio recommendations, samples were sequenced using the Illumina NovaSeq floor. Paired-end reads were appropriated to samples entrenched their special barcode and truncated by cutting off the barcode and primer sequence. They were then merged into paired-end reads using FLASH. According to fqtrim (v0.94), the quality filtering of the raw data is filtering under specific filtering conditions to obtain high-quality clean tags. Preconditioning the raw readings was done by using the following procedure: Cutadapt (v1.9), FLASH (v1.2.8), fqtrim, and Vsearch (v2.3.4) were used for data stitching and filtering (Ma et al., 2021a). DADA2 was called *via* qiime dada2 denoise-paired for length filtering and denoising (Caporaso et al., 2010). We obtained the feature table and feature sequence. Alpha diversity and Beta diversity analyses were conducted based on the feature sequence and feature table. Each group’s Venn diagram, PCoA diagram, heat map, stacked bar chart, and box diagram were displayed with R software (v3.4.4). According to the feature table, the SILVA (Release 138) database was used to annotate the species in the NT-16S database. The abundance of species in the sample was counted according to feature sequence. The LEfSe of each group was displayed with Nsegata-lefse (094f447691f0; Segata et al., 2011). Picrust2 function prediction was made using Picrust2 (v2.2.0b). We obtained the annotation results of database functions such as COG, EC, KO, PFAM, and TIGRFAM (Douglas et al., 2020). Bugbase phenotype prediction was made using Bugbase.^2^

## 3. Results

### 3.1. Sequencing data

**Figure 1:**
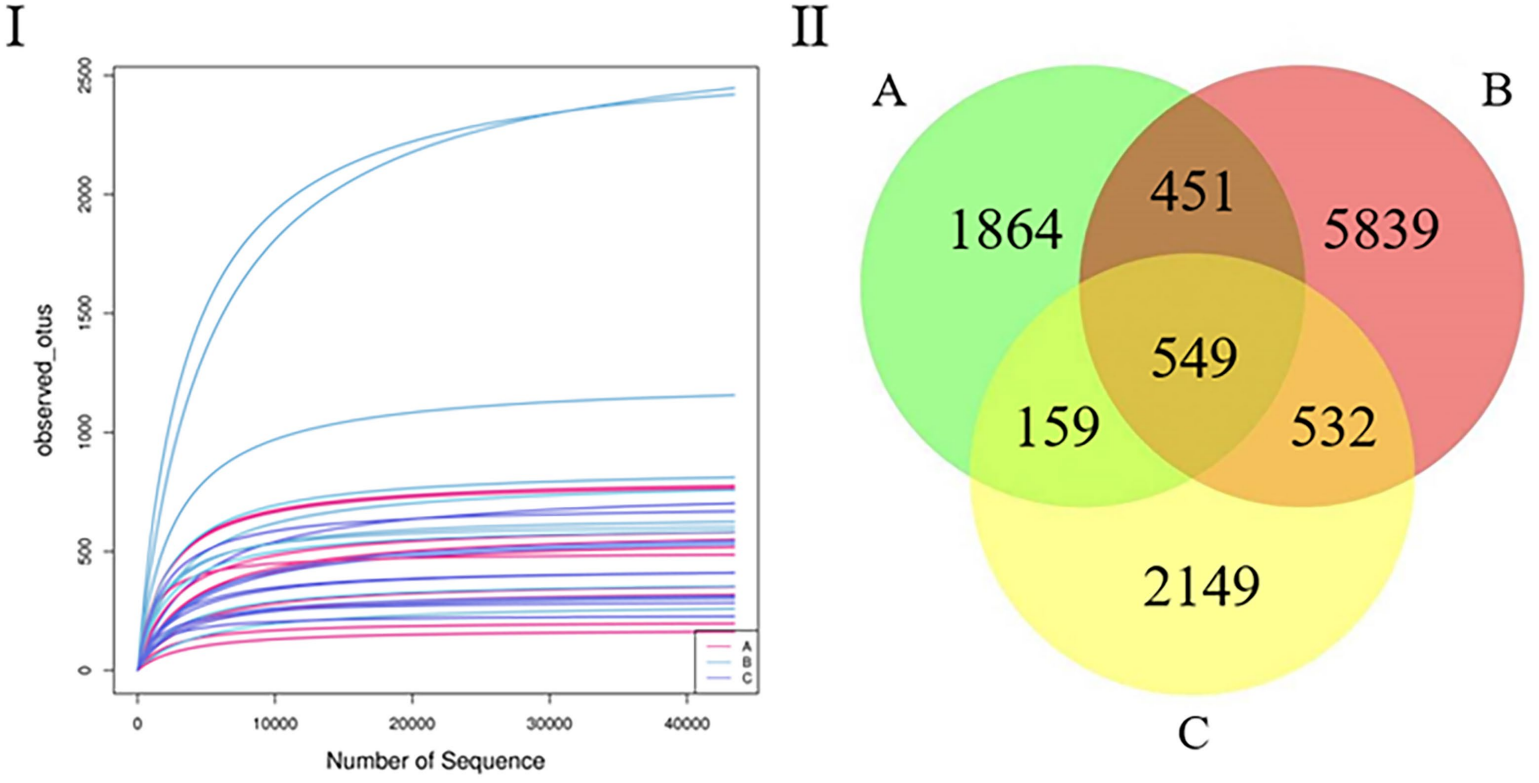
**(I)** Rarefaction curve. Curves of assorted colors show the observed OTUs (vertical axis) of each sample at a different number of sequences sampled (lateral axis). Flat curves indicate that sequencing depth is sufficient to reflect most of the microbial data in the samples. **(II)** Venn diagram. OTU distribution in the three groups.

After quality control, the samples yielded 1,376,048 sequences. The mean number of all sequences in all samples was 68,802. The sequencing quality was calculated using a rarefaction curve. All curves were flat, and the number of operational taxonomic units (OTUs) was close to saturation (Figure 1I). The rarefaction curves suggest that the sequencing amounts for all samples were sufficient to reflect most microbial diversity information. The total number of OTUs obtained was 11,543; groups A and B had 1,000 common OTUs. There were 1,864 and 5,839 unique OTUs in groups A and B, respectively. Groups B and C had 1,081 common OTUs, and there were 5,839 and 2,149 unique OTUs in groups B and C, respectively. Groups A and C had 708 common OTUs, and there were 1,864 and 2,149 unique OTUs in groups A and C, respectively (Figure 1II).

### 3.2. OTU abundance analysis

**Figure 2:**
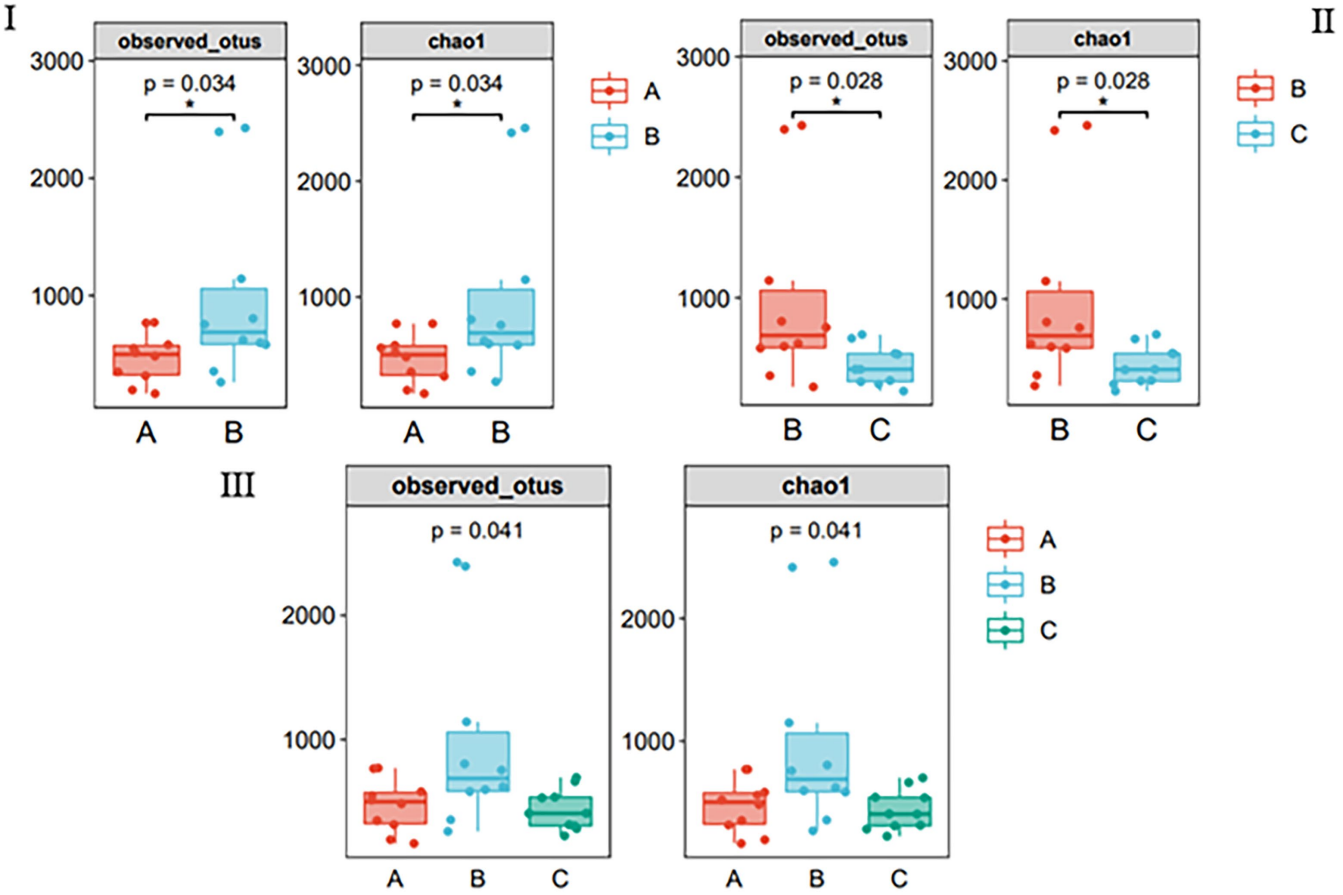
Species richness of gut bacteria of the wild *Arborophila rufipectus* and *Lophura nycthemera* measured *via* 16S rDNA sequencing (*p < 0.05). **(I)** Comparison of observed_otus and chao1 index between groups A and B. **(II)** Comparison of observed_otus and chao1 index between groups B and C. **(III)** Comparison of observed_otus and chao1 index between groups A, B, and C.

Multiple algorithms, such as observed_otus and Chao1, were used to estimate alpha diversity (Figures 2I–III). The results of the other algorithms are shown in Supplementary Table S1. For different species, the differences between samples in the same season were significant (Figure 2I); for the same species, there were significant differences between samples in different seasons (Figure 2II); however, there was no difference between the three groups (Figure 2III), and species richness in group B was significantly higher than that in other groups.

**Figure 3:**
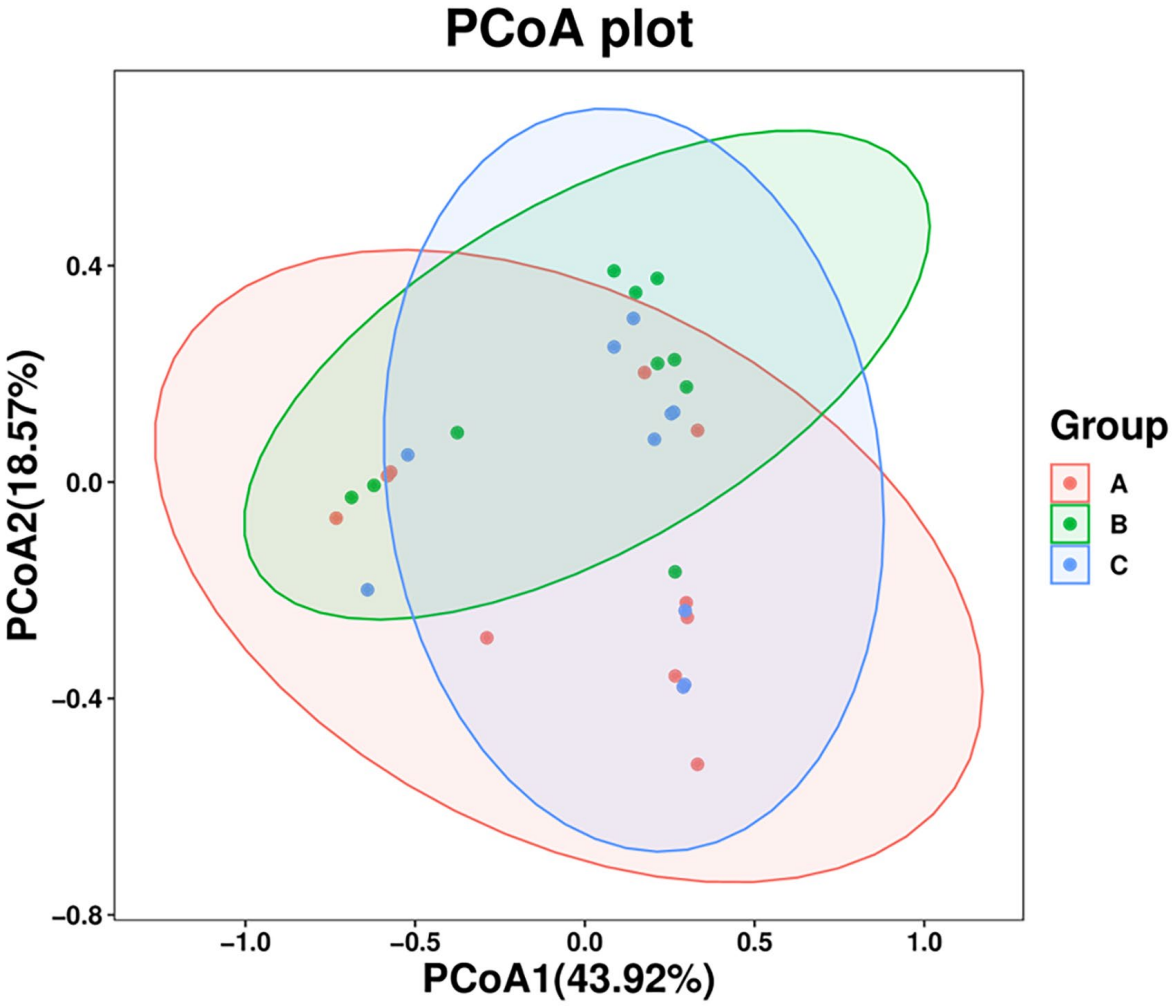
Principal coordinates analysis (PCoA) of wild *Arborophila rufipectus* and *Lophura nycthemera* gut microbial communities. A dot represents all samples, and distinct colors represent distinct groups.

The PCoA results showed that the three groups of stool samples did not cluster (Figure 3). This may be related to the fact that both *A. rufipectus* and *L. nycthemera* belong to Phasianidae. In contrast, the time interval between groups B and C was shorter. The UPGMA cluster number results also showed that most samples in the same group did not have allied branches (Supplementary Figure S1).

### 3.3. Community-composition analysis

**Figure 4:**
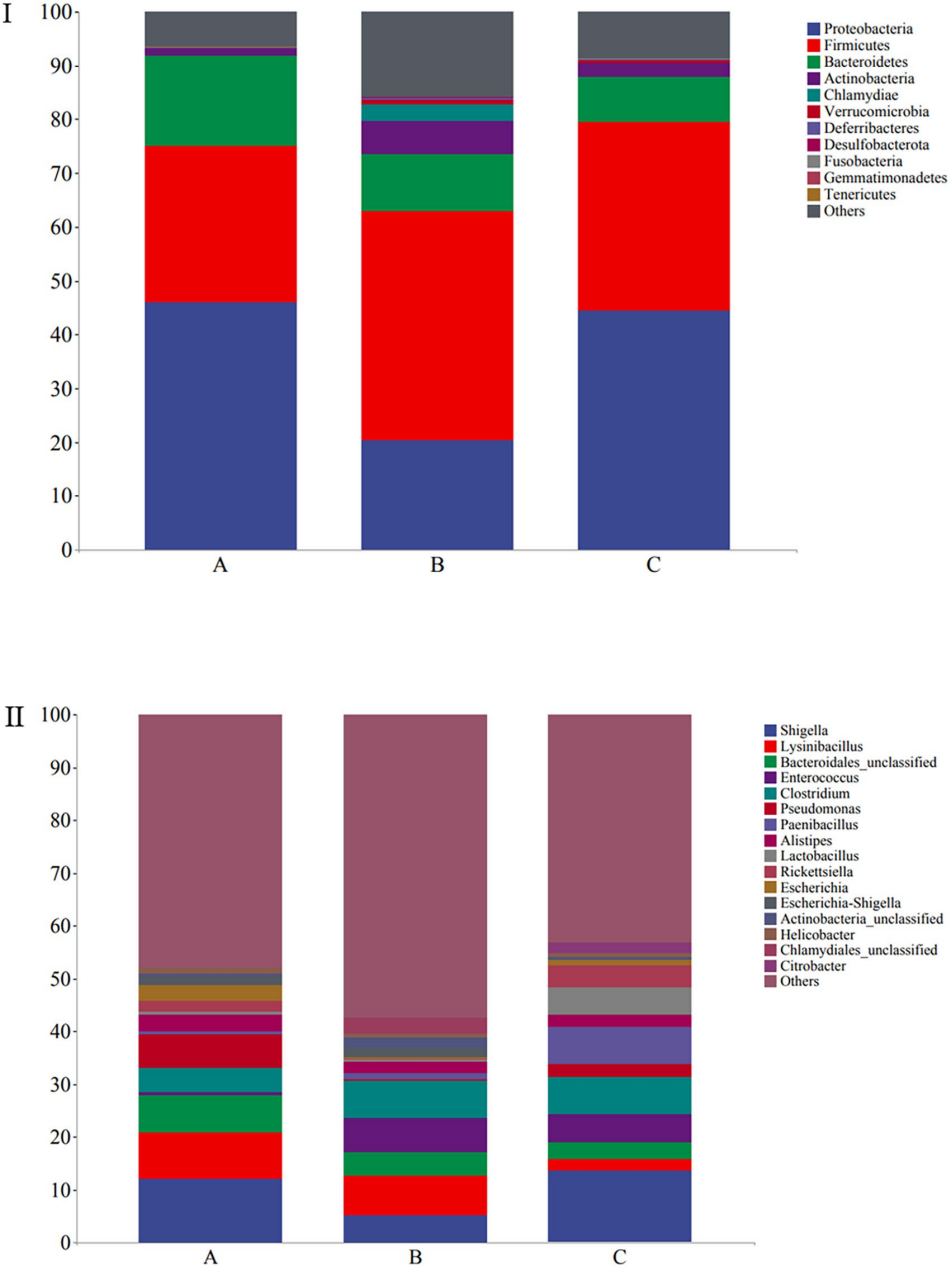
Stacked bar plots showing average percentages of the wild *Arborophila rufipectus* and *Lophura nycthemera* gut bacteria population compositions at the **(I)** phyla level and **(II)** genera level.

The microbial composition of the 30 samples was analyzed, and their mean relative abundance at the phylum level was calculated. The core phyla were: Proteobacteria (45.98%), Firmicutes (35.65%), Bacteroidetes (11.77%), and Actinobacteria (3.48%), accounting for 96.88% of the total microbial composition in all samples. Moreover, the core genera included *Shigella* (10.38%), *Lysinibacillus* (6.04%), *Bacteroidales_unclassified* (4.95%), *Enterococcus* (4.19%), and *Clostridium* (6.15%), with 16 core genera constituting 50.13% of the microbial composition identified in all samples (Figure 4I). At the phylum level, Proteobacteria (45.96%), Firmicutes (29.3%), Bacteroidetes (16.54%), and Actinobacteria (1.4%) were the predominant taxa (>1%) in group A. Firmicutes (42.64%), Proteobacteria (20.39%), Bacteroidetes (10.4%), Actinobacteria (6.31%), and Chlamydiae (3.1%) were the predominant taxa (>1%) in group B. Proteobacteria (44.58%), Firmicutes (34.97%), Bacteroidetes (8.36%), and Actinobacteria (2.74%) were the predominant taxa (>1%) in group C (Figure 4I).

At the genus level, *Shigella* (12.19%), *Lysinibacillus* (8.65%), *Acinetobacter* (7.88%), *Bacteroidales_unclassified* (7.2%), *Pseudomonas* (6.37%), *Clostridium* (4.53%), *Barnesiella* (3.33%), *Escherichia* (2.96%), *Escherichia-Shigella* (1.83%), and *Rickettsiella* (1.82%) were the predominant taxa (>1%) in group A. *Lysinibacillus* (7.37%), *Shigella* (5.28%), *Clostridium* (7.12%), *Enterococcuswere* (6.57%), *Bacteroidales_unclassified* (4.47%), *Escherichia-Shigella* (1.8%), *Barnesiella* (1.28%), and *Paenibacillus* (1.19%) were the predominant taxa (>1%) in group B. *Shigella* (13.67%), *Paenibacillus* (7.1%), *Clostridium* (6.83%), *Enterococcus* (5.46%), *Lactobacillus* (5.08%), *Rickettsiella* (4.15%) *Bacteroidales_unclassified* (3.2%), *Pseudomonas* (2.53%), *Lysinibacillus* (2.11%), *Citrobacter* (2.06%), *Escherichia-Shigella* (1.8%), and *Barnesiella* (1.28%) the predominant taxa (>1%) in group C (Figure 4II). A heatmap is provided in Supplementary Figure S1.

### 3.4. Bacterial isolation and identification

After the Community-Composition Analysis demonstrated that the dominant genus of *A. rufipectus* and *L. nycthemera* was *Shigella*, we cultured the bacteria in the feces, and *Shigella* was found. In total, five bacterial species were found (*Escherichia coli*, *Shigella*, *Enterococcus faecalis*, *Paenibacillus,* and *Citrobacter*). Due to the limitations of traditional bacterial culture techniques, *Clostridium, Rickettsiella*, and *Chlamydiales* could not be isolated. However, this also confirmed the existence of *Shigella* in the feces of these two species of wild birds. The evolution tree of bacteria is presented in Supplementary Figure S2.

### 3.5. LEfSe analysis

**Figure 5:**
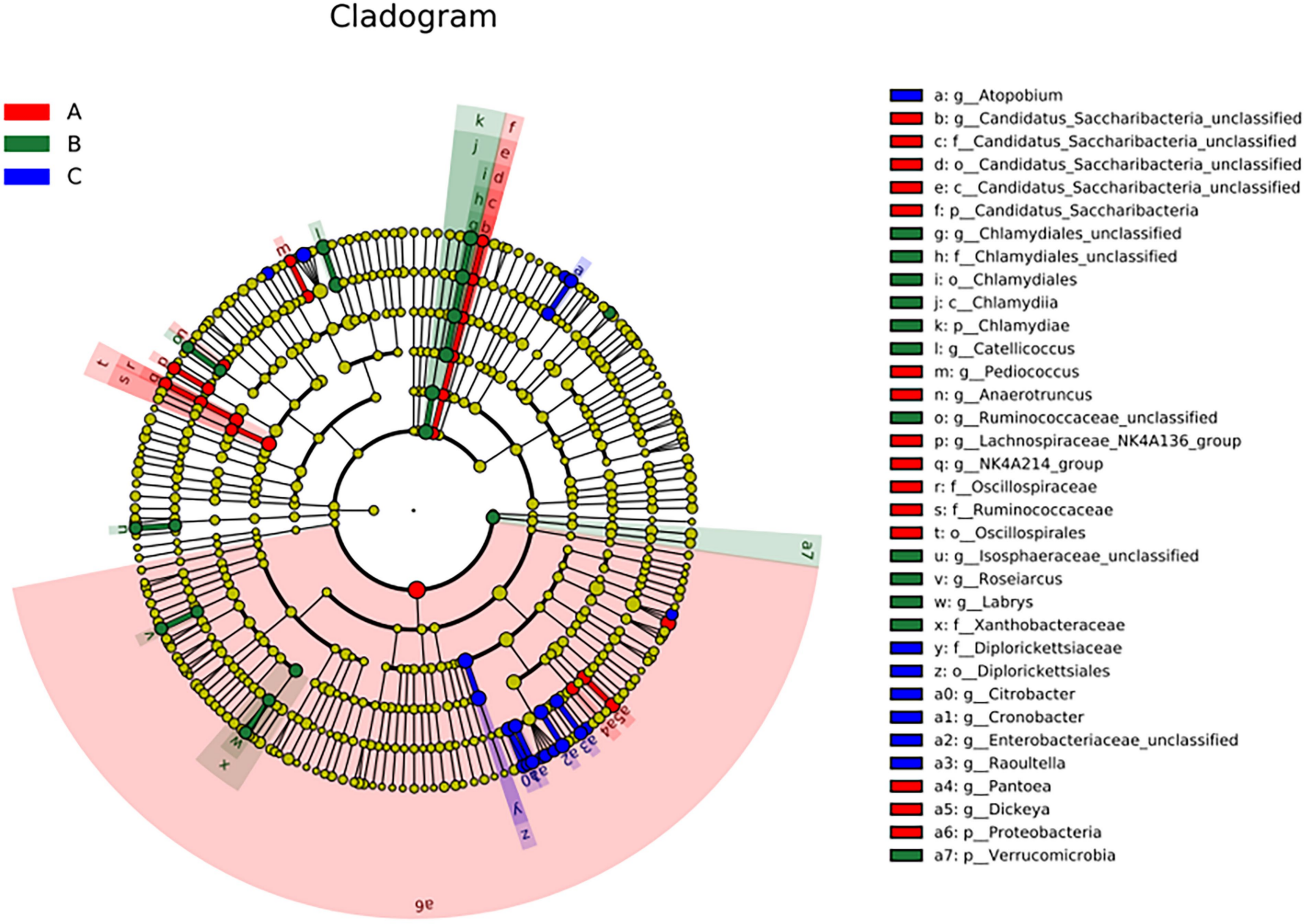
The linear discriminant fact size cluster trees of the three groups were used for 16S rRNA gene sequencing analysis. Distinct colors represent distinct groups; from the inside to the outside, the species level classification is shown: phylum, class, order, family, and genus.

**Figure 6:**
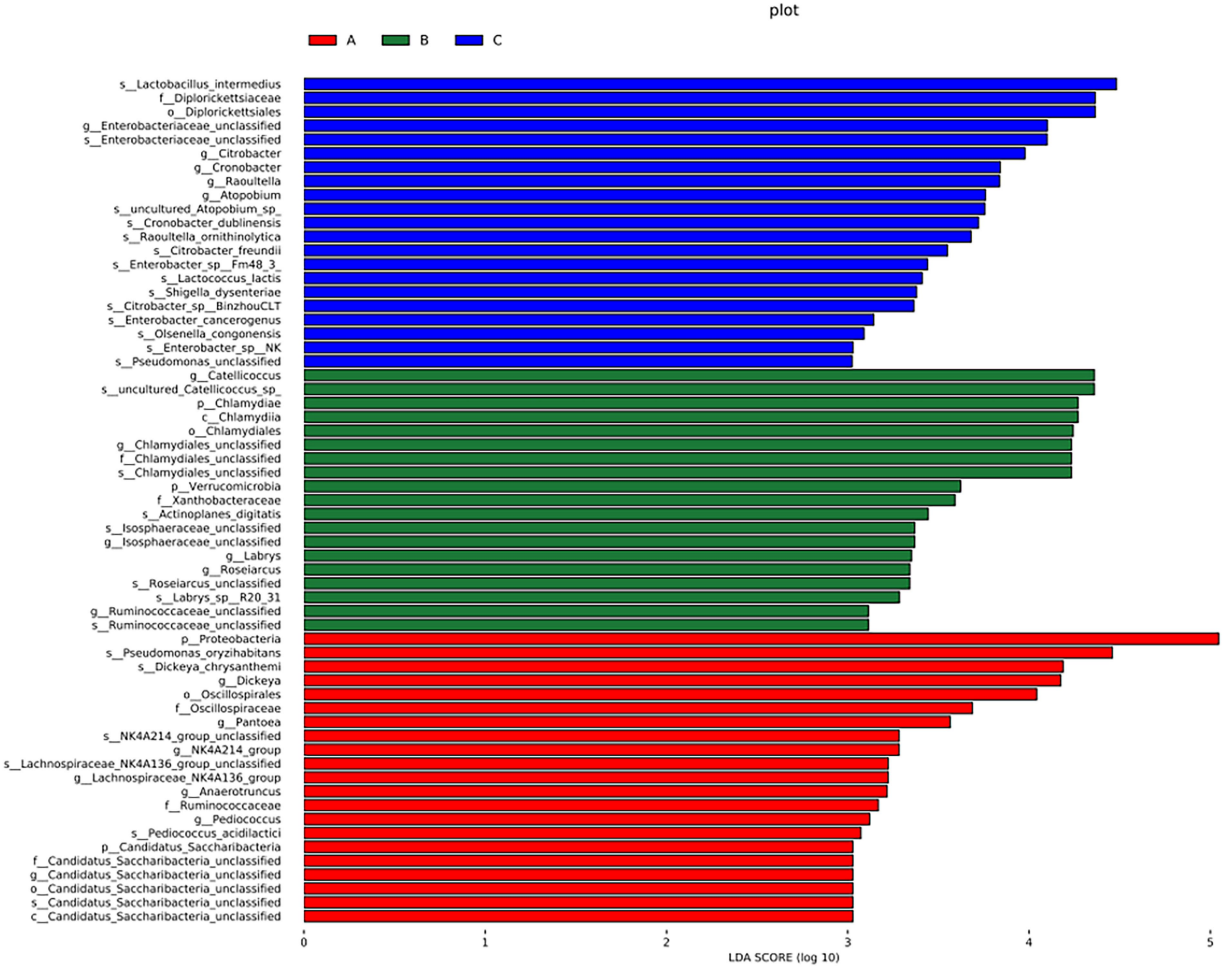
LDA demonstrated distinct bacterial genera enriched in the three groups. The color of the bar chart represents the higher abundance of different species in each group, and the length represents the influence degree of significant differences between different groups.

A cladogram of the LEfSe analysis is shown in Figure 5. The blue, green, and red areas represent groups A, B, and C, respectively. The blue, green, and red nodes in the cladogram denote the bacteria that play a pivotal role in groups A, B, and C, respectively. The yellow nodes correspond to bacteria that did not play a vital role in each group. The LDA indicated that LDA could determine the degree of influence of significantly varied species among different groups (Figure 6).

There were significant differences at the phylum level: Candidatus Saccharibacteria and Proteobacteria in the A group; Verrucomicrobla and Chlamydiae in the B group. There were also significant differences at the genus level: *Pantoea*, *Dickeya*, *Candidatus_Saccharibacteria_unclassified*, *Pediococcus*, *Anaerotruncus*, and *Proteobacteria* in the A group; *Chlamydiales_unclassified*, *Catellicoccus*, *Ruminococcaceae_unclassified*, *Labrys*, *llsosphaeraceae_unclassified*, *Roseiarcus*, and *Verrucomicrobia* in the B group; *Atopobium*, *Citrobacter*, *Cronobacter*, *Enterobacteriaceae_unclassified*, and *Raoultella* in the group C.

### 3.6. Analysis of potentially pathogenic bacteria

**Figure 7:**
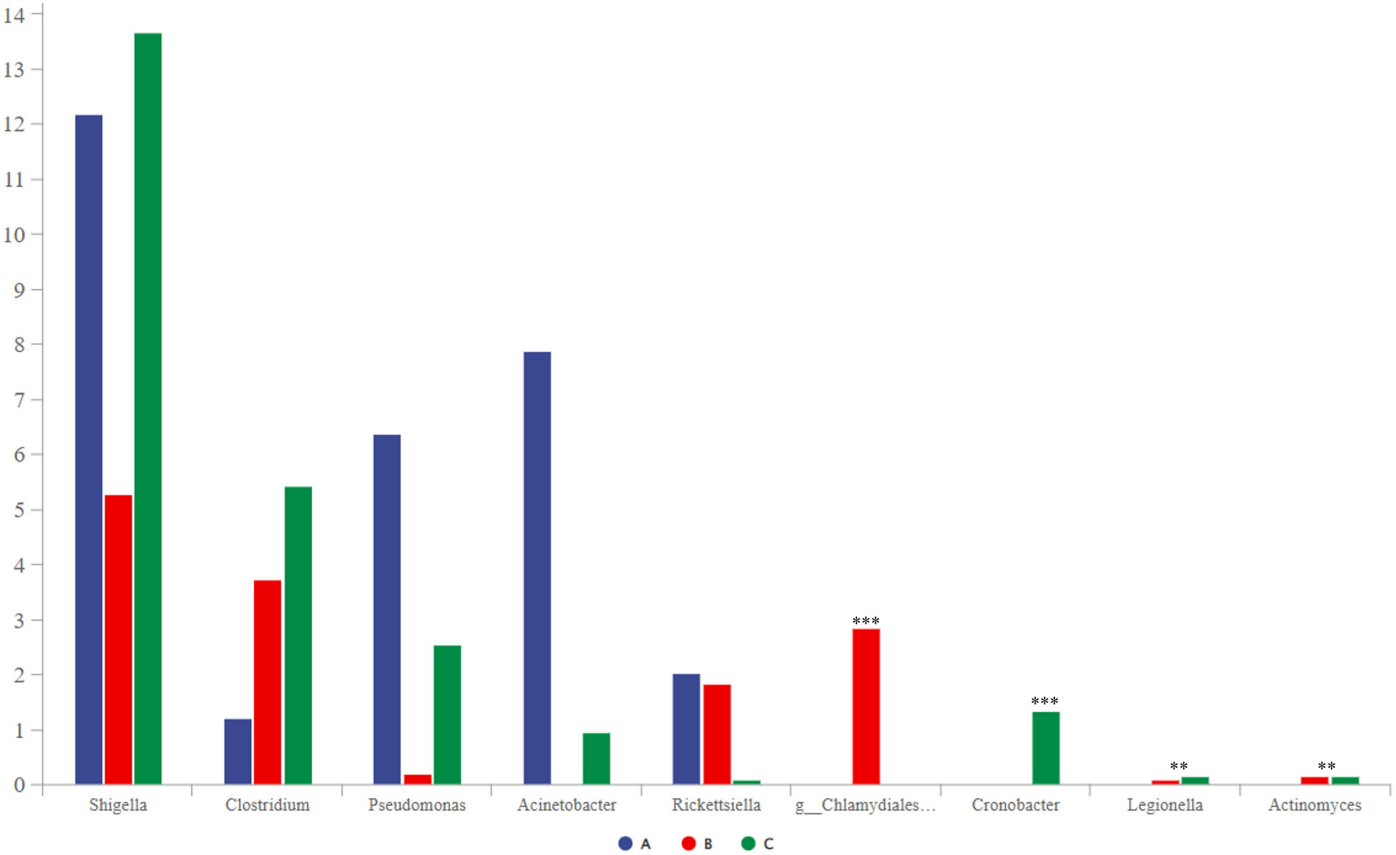
Comparative analysis of separate groups of pathogenic bacteria (*p* < 0.001, noted with “***”; 0.001 ≤ *p* ≤ 0.01, noted with “**”; 0.01 < *p* ≤ 0.05, noted with “*”; *p* > 0.05, unmarked).

Some potentially pathogenic bacteria, such as *Shigella*, *Clostridium*, *Pseudomonas*, and *Chlamydiales_unclassified*, were detected using community-composition analysis and LEfSe analysis. These bacteria can cause bird diseases (Shi et al., 2014; Knittler and Sachse, 2015; Uzal et al., 2016; Li et al., 2021); therefore, we decided to focus on them. *Shigella* was the most abundant, followed by *Clostridium*, *Pseudomonas*, and *Acinetobacter* (Figure 7). Among them, the abundance of *Chlamydiales_unclassified* was the highest in group B. *Cronobacter* only existed in group C, and group A did not contain *Legionella* or *Actinomyces*.

### 3.7. Predictive function gene analysis

**Figure 8:**
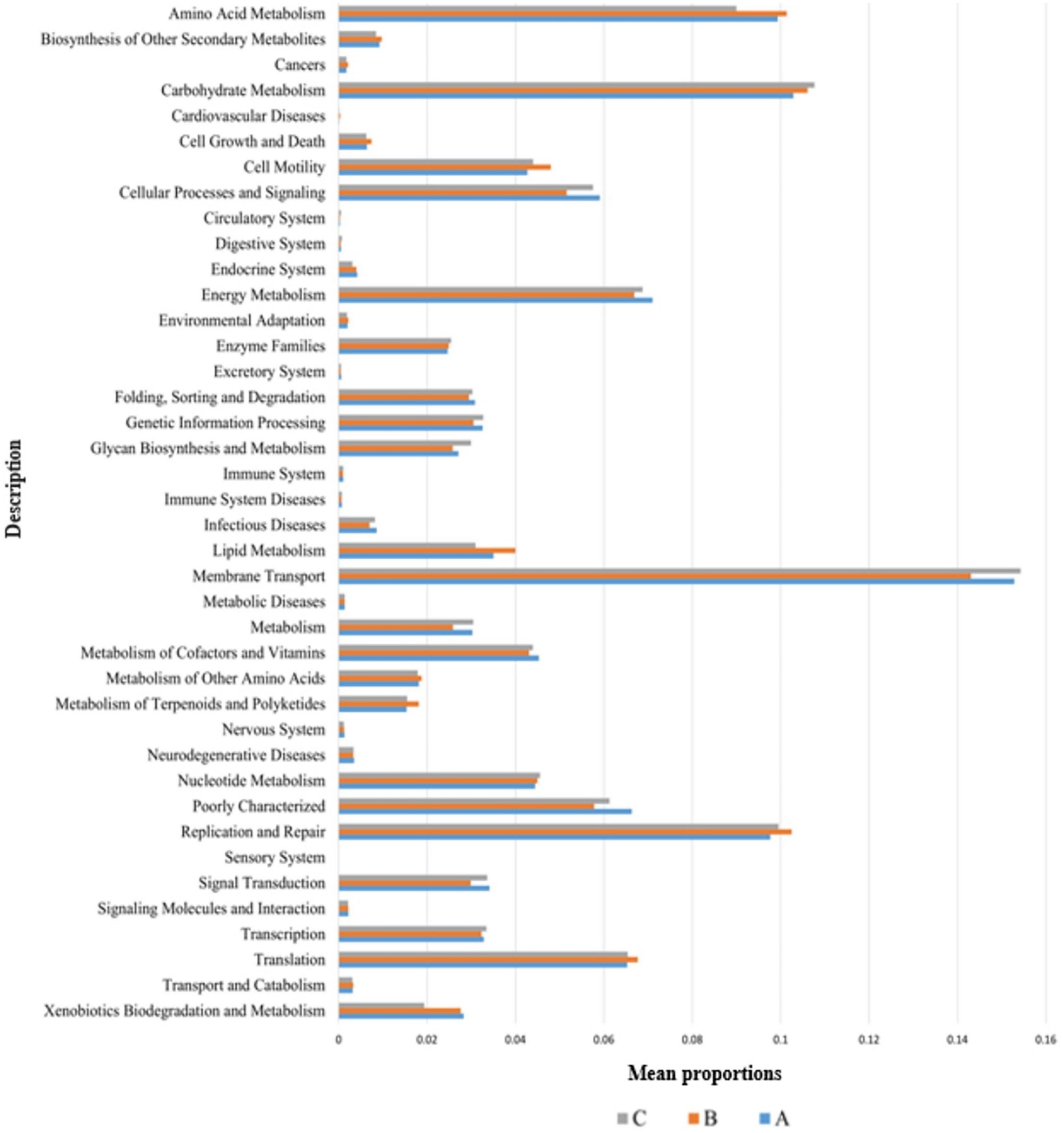
Wilcoxon test result map of pathway difference (KEGG level 2). The horizontal axis represents the relative abundance of each group, and the vertical axis represents the function of statistically significant differences. Abundance has no unit.

**Figure 9:**
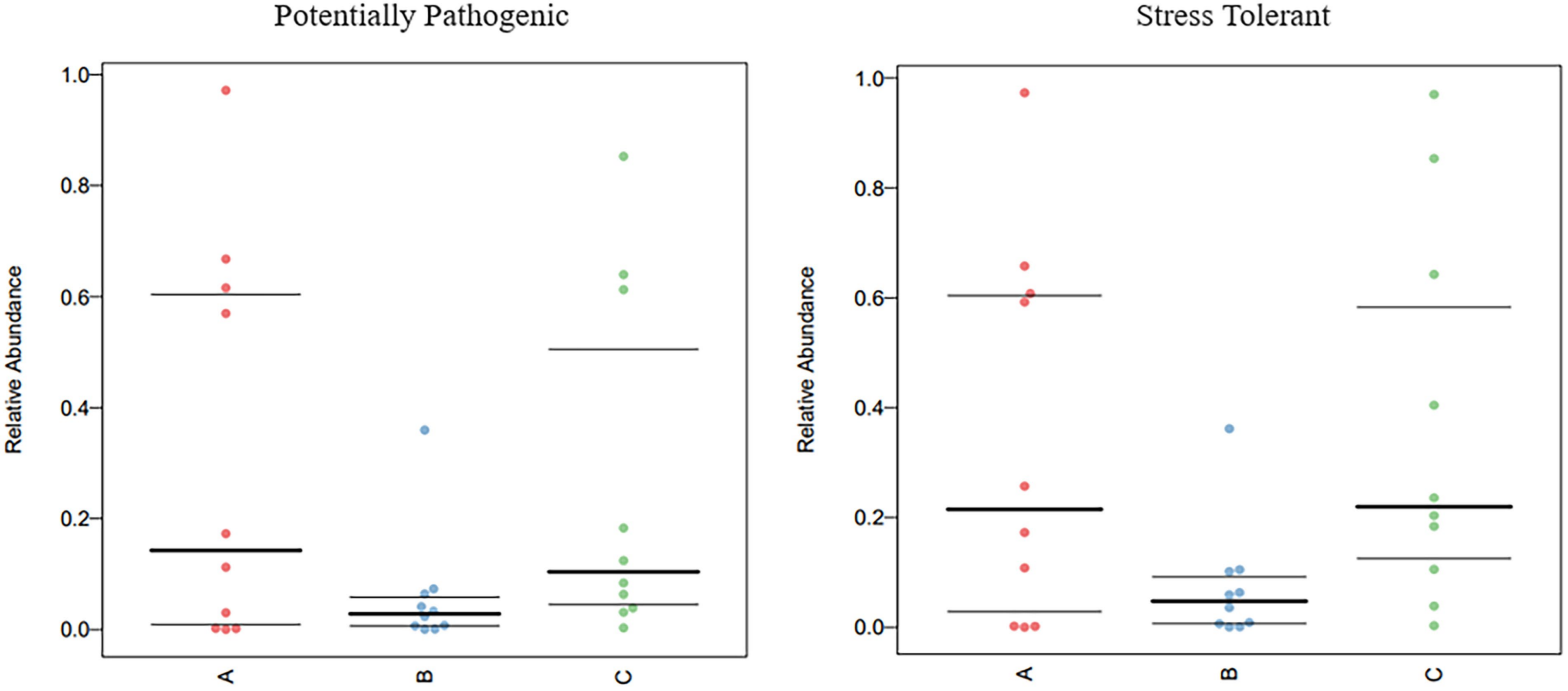
Comparison of bacterial phenotypes in samples. Scatter plots show the relative abundance of each sample in different phenotypes in different groups.

The functional genes of three groups of the samples were analyzed using the Genomes (KEGG) and Kyoto Encyclopedia of Genes database. There was little difference among the three groups, and membrane transport, carbohydrate metabolism, amino acid metabolism, and replication and repair accounted for the largest proportion. This is also consistent with the results of the principal coordinate analysis. However, genes related to cell growth and death were detected, which is generally related to pathogenic bacterial infection, indicating that groups A, B, and C may have been infected with pathogenic bacteria (Figure 8). According to the prediction of bacterial phenotype, the abundance of potentially pathogenic and stress-tolerant bacteria in group B was the lowest (*p* < 0.05; Figure 9); other predictions of the bacterial phenotypes are shown in Supplementary Figure S3.

## 4. Discussion

*Arborophila rufipectus* is one of the rarest *Arborophila* species worldwide, with approximately 2,200 individuals distributed in China. China has implemented many conservation strategies, such as establishing nature reserves and captive breeding, to protect the habitat and populations of *A. rufipectus*. Nonetheless, these strategies have overlooked the effect of the gut microbiome in wild *A. rufipectus*. In this study, we analyzed the gut microbiota of *A. rufipectus* for two consecutive seasons by analyzing the 16S rRNA gene sequences.

The rarefaction curve precisely reflects the justifiability of the sequencing data and indirectly reflects the enrichment of species in the sample. When the curve tends to be flat, the quantity of sequencing data is gradually reasonable (Ma et al., 2021a), which displays high evenness among samples, showing that the sample size was sufficient and that data analysis could be performed (Torok et al., 2011; Ma et al., 2021b).

Alpha diversity analysis was used to analyze the species diversity of independent samples. In terms of season, there was a significant difference in the Chao1, observed_otus, Simpson, and Shannon indices (*p* < 0.05) between the B and C groups. Regarding seasons, there was a significant difference in the Chao1, observed_otus, and indices (*p* < 0.05) between groups A and B. The abundance of group B was the highest, indicating that the species richness and diversity of group B were the highest among the three groups. It can also be seen that the OTU of group B is the highest in the Venn diagram (Figure 1I); this phenomenon may be related to the season and diet (Lu et al., 2021).

High-throughput 16S rRNA sequencing analysis allowed the gut microbial composition of wild *A. rufipectus* to be explored without culture (Yue et al., 2021). The gut microbiotas of wild *A. rufipectus* and *L. nycthemera* were mostly composed of Proteobacteria, Firmicutes, Bacteroidetes, and Actinobacteria at the phylum level, accounting for 96.88% of the total community. This corresponds with the formerly described core gut microbial communities in wild birds (Waite and Taylor, 2014; Spergser et al., 2018). The presence of these four bacterial taxa in the gut microbiota indicated that these bacteria were probably associated with crucial host gut functions such as immune response, metabolism, and nutrient digestion. The study found that higher ratios of Firmicutes and Proteobacteria were always linked with diets embracing plant ingredients (Rimoldi et al., 2018), which could help *A. rufipectus* to digest cellulose and hemicellulose (Morrison et al., 2009).

A total of 16 dominant genera were identified at the genus level. *Shigella* represented the highest percentage among these genera, which is quite different from previous studies. In other studies, *Lactobacillus* was the most abundant at the genus level in wild birds (Wang et al., 2019; Gao et al., 2021). However, the proportion of *Lactobacillus* was only 2.07% in the samples collected in our study. *Lactobacillus* is considered a healthy gut bacterium because of its ability to activate gut development, digestive function, and immune responses (Ringø et al., 2018). *Shigella* is a considerably infectious enteropathogen that invades and elicits deep inflammation and tissue damage in the colorectal epithelium (Schroeder and Hilbi, 2008; Carayol and Tran Van Nhieu, 2013).

One study used *Shigella* isolated from humans to infect three-day-old SPF chickens and found that intraperitoneal injection could cause death in SPF chickens (Shi et al., 2014). This indicates that *Shigella* can infect humans and chickens, which is of public health significance. We also detected other potentially pathogenic bacteria, such as *Pseudomonas*, *Clostridium, Rickettsiella*, and *Chlamydiales_unclassified*. The specific species and genus of Chlamydiales have not been identified, and birds are known to be the elementary host for three Chlamydial species: *Chlamydia psittaci*, *Chlamydia gallinacea*, and *Chlamydia avium* (Laroucau et al., 2008). *C. psittaci* has been known for decades as the causative agent of avian chlamydiosis in psittacine birds and humans (Andersen, 1991; Geigenfeind and Haag-Wackernagel, 2010). Recent research has reported *C. psittaci* infections in turkeys, ducks, pigeons, and geese (Van Loock et al., 2005; Cong et al., 2013; Ling et al., 2015; Zhao et al., 2015). In the bacterial culture isolated from the fecal sample, *Shigella* was found; however, due to the limitations of traditional culture methods, we were unable to isolate *Chlamydia*. Unlike other species, since birds have a cloaca, fecal flora does not fully represent intestinal flora, but some potentially pathogenic bacteria have been found when using high-throughput sequencing. The isolation of potentially pathogenic bacteria in feces does not indicate the health status of the host. The prevalence of these pathogens in wild birds is largely unknown and may be a source of zoonotic infections. The present data advise that appropriate measures should be implemented to eliminate the spread of diseases *via* birds.

Potentially pathogenic bacteria tend to have certain levels of infectivity when the immune system in the animal’s body is compromised. Groups A and B contained different species, but *Shigella* was also found in group A, similar to other potentially pathogenic bacteria. These results indicate that these wild birds in the reserve carry the opportunistic pathogen. Under certain conditions, particularly when the immune system is negatively affected by various stress factors such as environmental changes, the risk of infection increases among different bird species. However, the microbial ecology in feces cannot accurately reflect the health status of the host as some animal pathogens do not cause any diseases in birds. On the one hand, we did not perform targeted etiological research to confirm the pathogenesis of these potential pathogens. On the other hand, we could not legally capture these birds for further research and verification. Therefore, we can only put forward this point of view to provide a reference for the scientific conservation of wild birds such as *A. rufipectus*.

In summary, we used high-throughput sequencing to analyze the gut microbiome of *A. rufipectus* and used *L. nycthemera* from the same reserve as the species control group. The composition, diversity, and function of the gut microbiome are related to that of *A. rufipectus*. Several potential pathogens were identified in the gut microbiota of *A. rufipectus* and *L. nycthemera*, which can serve as a warning sign for protecting these endangered species. However, considering the small sample size and large inter-individual variance, it is evident that fecal samples do not accurately reflect the health status of the host and that wild birds cannot be captured to observe clinical symptoms. Future research should sample *A. rufipectus* more widely in time and space for more detailed comparative analyses. Our study is the first step toward describing the gut microbiome in wild *A. rufipectus*.

## Data Availability Statement

The datasets presented in this study can be found in online repositories. The names of the repository/repositories and accession number(s) can be found in the article/Supplementary material.

## Ethics Statement

The animal study was reviewed and approved by Sichuan Agricultural University Animal Ethics Committee and Sichuan Laojunshan Mountain National Nature Reserve (permit number DYYS20210329).

## Author contributions

XM, JL, BC, XL, ZL, SF, and SC carried out the sample collections, conceived the study, and drafted the manuscript. JL, SC, ZZu, JD, XH, DC, YWa, and QZ participated in the data analysis. ZZh, YWe, GP, YJ, and YG participated in the study design and coordination and helped draft the manuscript. All authors contributed to the article and approved the submitted version.

## Funding

This study was supported by the Investigation on the pathogen of *Arborophila rufipectus* of Laojun Mountain National Nature Reserve (CDXZ-2021-0307-1).

## Conflict of interest

The authors declare that the research was conducted in the absence of any commercial or financial relationships that could be construed as a potential conflict of interest.

## Publisher’s note

All claims expressed in this article are solely those of the authors and do not necessarily represent those of their affiliated organizations, or those of the publisher, the editors and the reviewers. Any product that may be evaluated in this article, or claim that may be made by its manufacturer, is not guaranteed or endorsed by the publisher.

## Footnotes

^1^ http://www.ncbi.nlm.nih.gov/blast

^2^ https://github.com/knig-Hts-lab/BugBase
