# Supplementary material for: Analysis of microbial diversity in the feces of Arborophila rufipectus
Source: Front Microbiol. 2023 Feb 2;13:1075041. doi: 10.3389/fmicb.2022.1075041 (PMC9932278; doi:10.3389/fmicb.2022.1075041)
Supplement: Supplementary file 1 [file Data_Sheet_1.PDF]

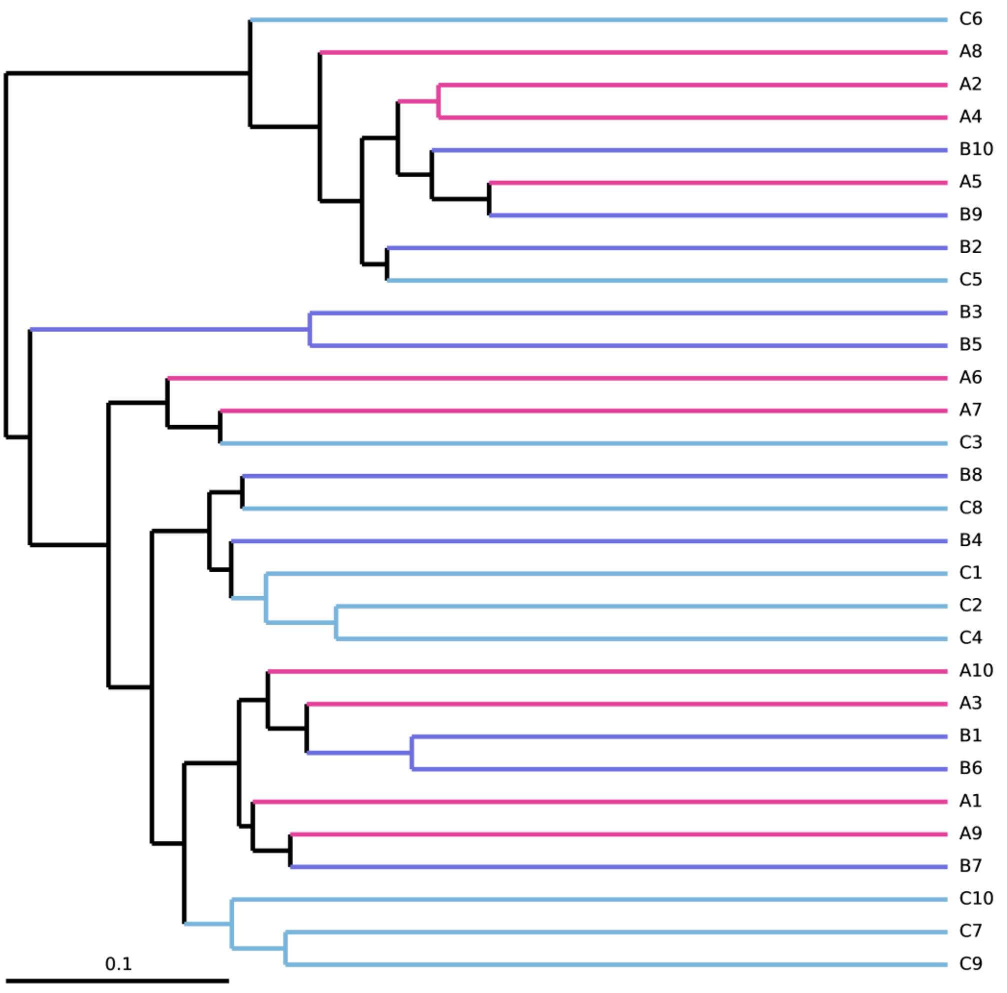

Fig S1 Weighted uniFrac UPGMA cluster

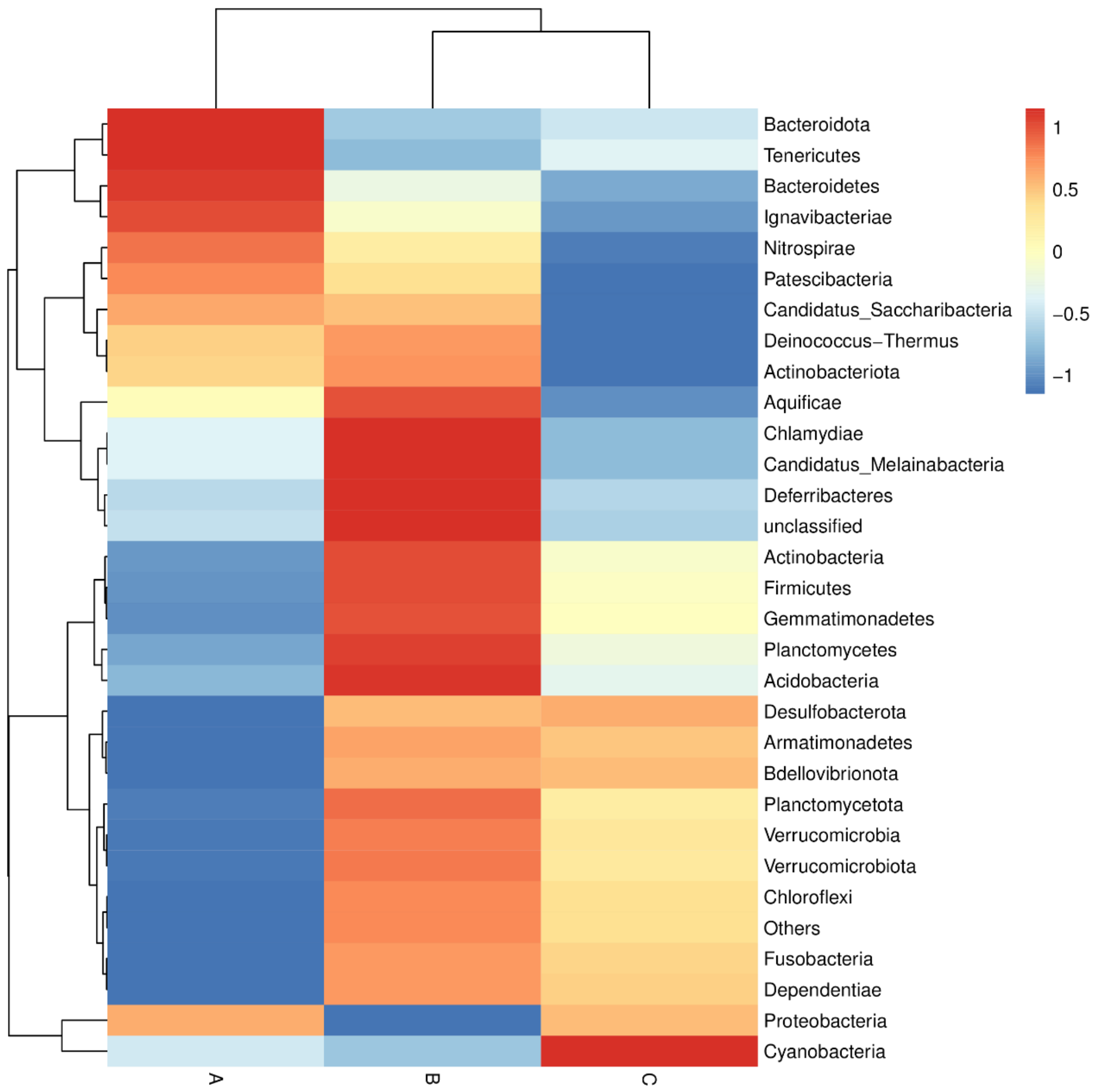

Fig S2 Heatmap showing the relative abundance of season-related phylum.

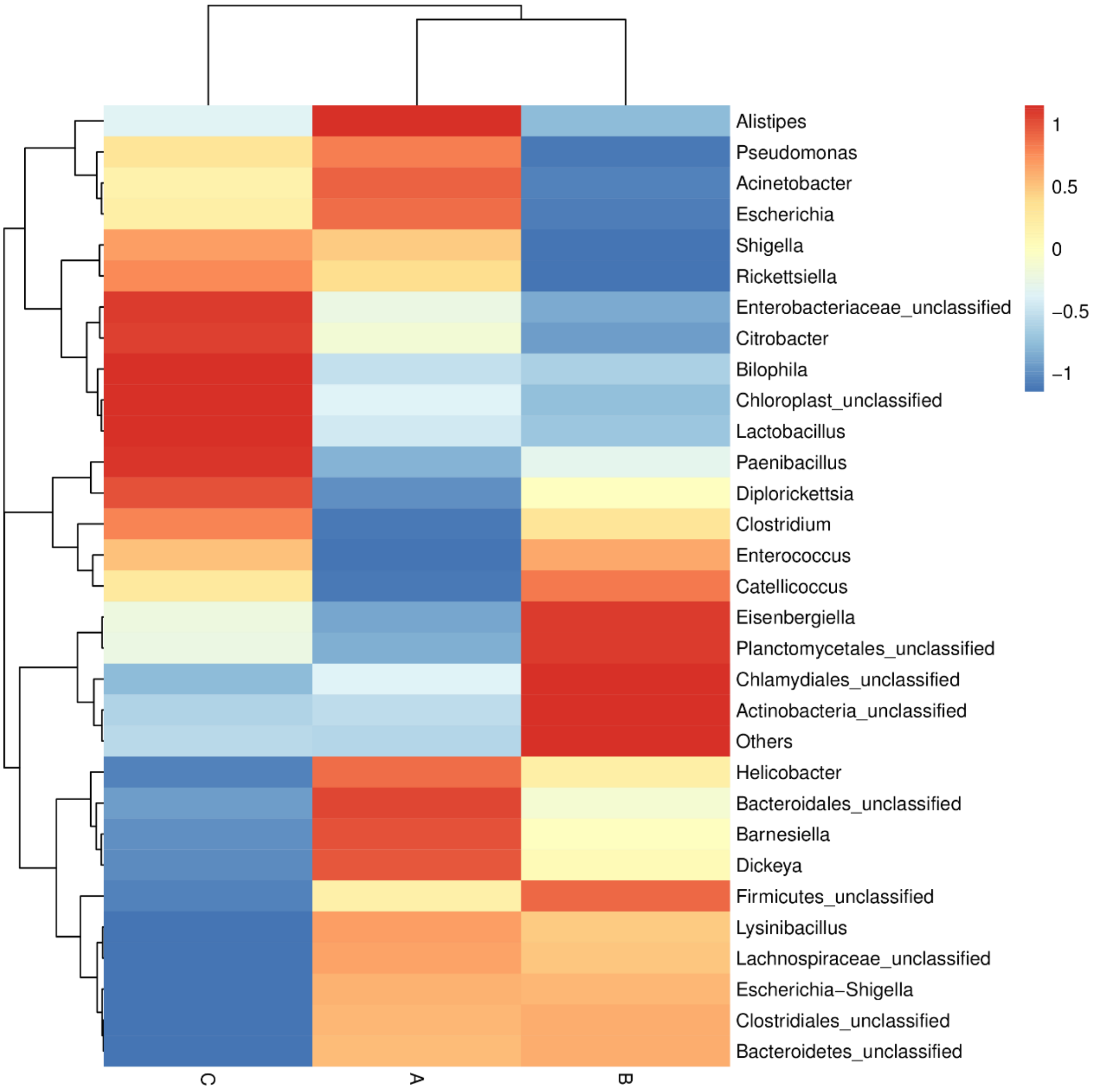

Fig S2 Heatmap showing the relative abundance of season-related genera.

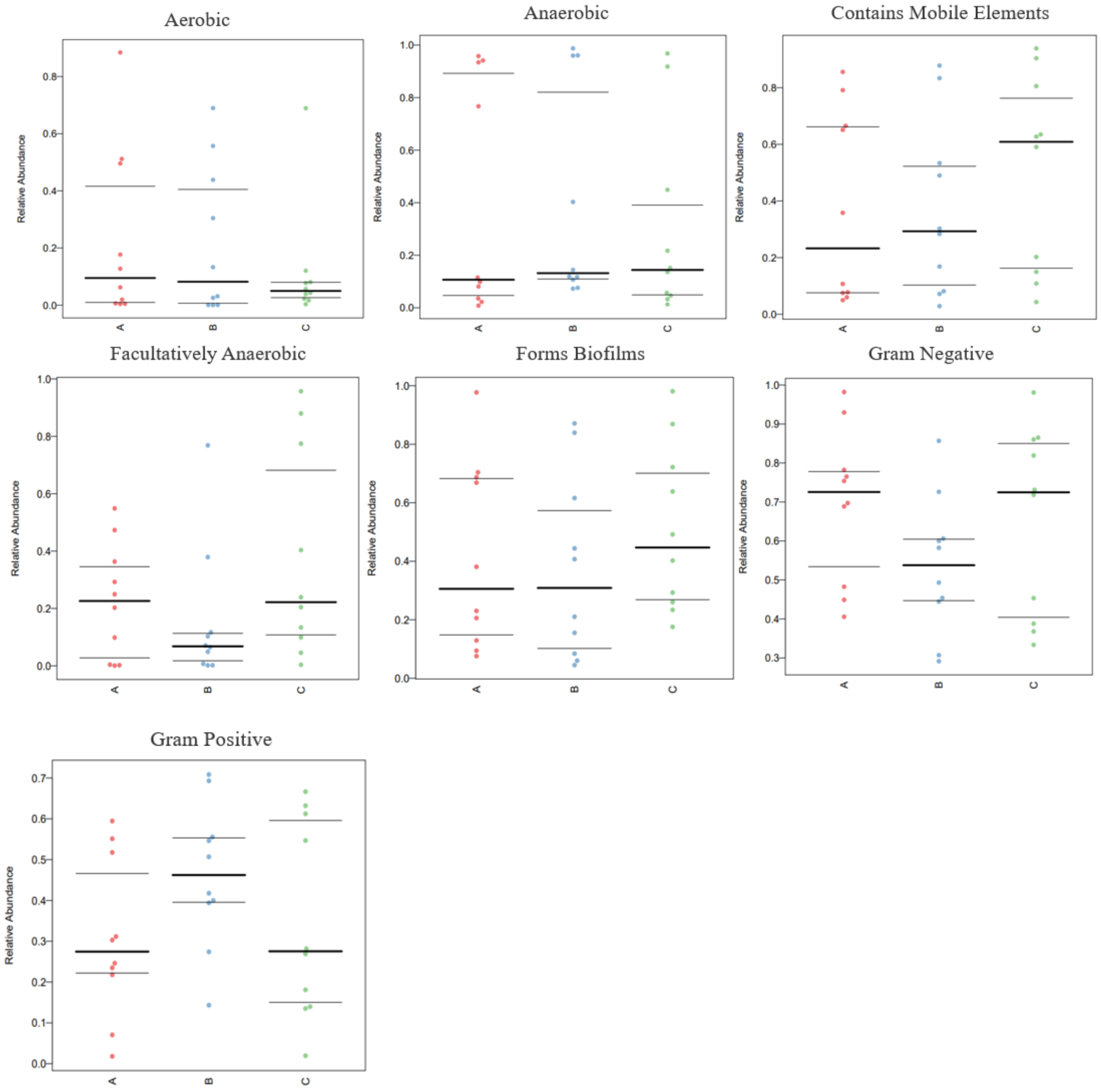

Fig S3 Comparison of bacterial phenotypes in samples.
